# COVID-19 Pandemic Management Strategies and Outcomes in East Asia and the Western World: The Scientific State, Democratic Ideology, and Social Behavior

**DOI:** 10.3389/fsoc.2020.575588

**Published:** 2020-11-26

**Authors:** Hang Kei Ho

**Affiliations:** Faculty of Social Sciences, University of Helsinki, Helsinki, Finland

**Keywords:** COVID-19, East Asian scientific state, facemask, herd immunity, pandemic strategy, public health, SARS, social behavior

## Introduction

In December 2019, a new strain of Coronavirus, later identified as SARS-CoV-2 (Severe Acute Respiratory Syndrome Coronavirus 2) or COVID-19, began to surface in Wuhan, China. By February 2020, COVID-19 had become a pandemic which required emergency measures across the globe. However, regions in East Asia and Europe adopted different pandemic strategies which led to different outcomes. This article approaches different public health strategies, from both natural science and social science perspectives through the lens of East Asia and the Western world in three ways. First, using peer-reviewed scientific literature from the fields of infectious disease, medicine and public health, it examines how Hong Kong and its medical community dealt with the SARS outbreak in 2003 and the COVID-19 pandemic in 2020. This is important because Hong Kong has been acknowledged by the scientific community for having the most effective COVID-19 strategy (Gibney, 2020). Second, using scientific literature as a point of departure, it argues that Hong Kong, Taiwan, Singapore and South Korea (known as the Four Asian Tigers or East Asian Miracle) excel in STEM (science, technology, engineering, and mathematics) subjects that provide citizens with the skills to understand the science behind dealing with COVID-19. This article introduces the concept of the “scientific state” to capture such observation which goes beyond the popular belief that East Asian societies are better at obeying pandemic strategies set out by authorities in comparison to the West. Third, and building on the last point, it explores how social norms play an important role in dealing with the pandemic. The article concludes by arguing that an attitude of Anglo-European exceptionalism meant that successful strategies in the East were overlooked and led to undesired outcomes from Western management of the pandemic. Nonetheless, regions in East Asia did extremely well in containing COVID-19 not because of citizens were obedient to undemocratic pandemic management rules, but competent medically trained government ministers set out rules which citizen with high STEM proficiency understood and respected.

## Management Strategies of SARS and COVID-19: A Scientific Approach In East Asia

After the SARS outbreak in Hong Kong that originated in mainland China in 2003, measures were drawn up by experts that included the introduction of preventive education and publicity, tracing the source of infection, isolating and carrying out surveillance of contacts, closing educational institutions, checking body temperature at borders, deploying public cleansing campaigns and conducting diagnostic tests (Lee, 2003). Significantly, strategies used for containing SARS can also be used for COVID-19 to reduce cases and deaths (Wilder-Smith et al., 2020). Hence, in the absence of pharmaceutical interventions, Hong Kong managed to reduce the COVID-19 transmission rate by influencing public behaviors without lockdowns (Cowling et al., 2020). Similarly, rapid widespread testing and contact tracing strategies in South Korea were effective in containing the COVID-19 virus without lockdown (Lee and Lee, 2020). Nonetheless, implementing such strategies requires political will to ensure that the “short-term cost of containment will be far lower than the long-term cost of non-containment” (Wilder-Smith et al., 2020).

Since the SARS outbreak, usage of facemasks by the wider population has been adopted in Hong Kong and other East Asian regions. Research suggests that widespread use of surgical masks and N95 respirators is beneficial when worn properly and used alongside other strategies to control outbreaks (Chan and Yuen, 2020). Furthermore, since Coronavirus respiratory droplets and aerosols are exhaled when breathing and coughing, “surgical face masks could prevent transmission of human coronaviruses and influenza viruses from symptomatic individuals” (Leung et al., 2020). Additionally, since SARS-CoV-2 enters through the nose as well as other mucus membranes, the widespread use of masks can “prevent aerosol, large droplet, and/or mechanical exposure to the nasal passages” (Hou et al., 2020)^1^. Because of the widespread use of facemasks, over the past 5 years Hong Kong has experienced the shortest winter surge of seasonal influenza-−5 weeks rather than the typical 12 to 18 week period (Chan and Yuen, 2020). Further statistical analysis shows that with 96.6 per cent of the population wearing facemasks in Hong Kong, confirmed COVID-19 cases were extremely low at 129.0 per million population (Cheng et al., 2020). This is significant because Hong Kong has the third highest population density in the world (7,140 per km^2^) and is in close proximity to the original Chinese epicenter of the outbreak. Despite this, the fact that those populations experienced SARS has allowed them to become more aware of the risks posed by the current COVID-19 pandemic which resulted in preventive and protective measures being taken more seriously (Wilder-Smith et al., 2020).

## Using An Ideology To Solve A Medical Problem: East Asian “Scientific State” VS. Western Democracy

Some commentators argue that draconian policies supposed to control the spread of the virus tend to make the pandemic worse (Giuffrida and Cochrane, 2020), and that democratic governments are better at controlling the virus (Ben-Ami, 2020). However, this section argues that COVID-19 should be seen as a scientific problem rather than a political one.

A crisis such as COVID-19 highlights some Western government officials might not have taken scientific knowledge nor the pandemic seriously because they tend to be made up of career politicians such as the UK who have narrow occupational background and lack life experiences (Allen et al., 2020) but the opposite trend can be observed in Eastern governments. For example, the current Secretary of State for Health of the United Kingdom Matt Hancock does not have a background in medicine, nor does his predecessor Jeremy Hunt. In contrast, Sophia Chan, the current Secretary for Food and Health in Hong Kong was a professor in nursing and consultant to the World Health Organization (WHO). Moreover, Chen Shih-chung, the current Ministry of Health and Welfare in Taiwan was medically trained. As well, the former Vice President of Taiwan Chen Chien-jen is an epidemiologist. Furthermore, Park Neung-hoo, the current Minister of Health and Welfare in South Korea was a professor in social welfare. These countries have not experienced the problems of Western policies that have failed to check the spread of the virus and resulted in high death rates per million population:^2^ Belgium 987; Denmark 124; France 560; Finland 65; Germany 125; Italy 634; Netherlands 428; Norway 52; Spain 767; Sweden 587; Switzerland 262; UK 680; US 709. In contrast, far fewer deaths per million population were reported in many South East Asian regions: Hong Kong 14; Japan 14; Macau 0; Malaysia 8; Singapore 5; South Korea 9; Taiwan 0.3; Thailand 0.8, Vietnam 0.4. This appears to be because they have been prepared for pandemics with excellent healthcare and warning systems. Furthermore, testing capacity has tended to be low in Europe and since there were no posthumous tests (Giugliano, 2020) that would uncover more cases than currently identified in death certificates, the actual rates of infection and deaths may be significantly higher than reported (Burn-Murdoch et al., 2020).

There is also an assumption in the West that populations in the East are Confucian and undemocratic, and therefore tend to be more obedient to rules. Perhaps Confucian ideas can be identified in some East Asian regions but Hong Kong, Japan, Singapore, South Korea, Taiwan, and so on have different forms of democracy. Most importantly, this article argues that the difference has more to do with governments of East Asia's “scientific states” for two reasons. First, these countries produced the most highly educated populations in the world; seven out of ten best performing countries / economies^3^ in terms of mathematics and science are located in East Asia (OECD, 2019). Second, their populations value scientific leadership and excel in the STEM fields which are key to understanding the science of the pandemic.

It is evident that citizens of the East do not follow policies which are seen to be ineffective. For instance, in January 2020, despite the number of imported cases continuing to increase in Hong Kong, the government did not close its borders with mainland China (Chan, 2020). But in February, when thousands of doctors and nurses went on strike, the government reversed its decision.

Moreover, despite the proven effectiveness of the widespread use of facemasks, the WHO and some governments, such as the UK, initially did not recommend it. There are at least four reasons for this. First, a global shortage of personal protective equipment (PPE) affected availability and guarantee supply to the public could not be guaranteed (Pickard and Asgari, 2020). Moreover, although the US Centers for Disease Control and Prevention (CDC) recommended their citizens should consider the use of face coverings from April 2020 (Fisher et al., 2020), it was left up to each US State to decide on how such recommendation should be adopted as policy and implemented. In addition, some countries such as Finland (Ministry of Social Affairs Health, 2020) and the Netherlands (Reuters, 2020) initially did not recommend the use of facemasks based on their own scientific experts' opinion, but later reversed the decision. And some countries such as Sweden did not recommend the use of facemasks at all (Milne, 2020a). Problematically, since most people infected with the virus are asymptomatic or only show mild symptoms (Randolph and Barreiro, 2020), they often cause the virus to spread without realizing.

Second, many Western governments have been concerned with the wider implications for civil rights because a “mask mandates use the coercive power of the state to require a person to do something that they would otherwise not choose to do” (Blunt, 2020).

Third, some governments argued that wearing facemasks might be a source of “social stigma and discrimination against those who do not wear one” (Royo-Bordonada et al., 2020).

Fourth, some countries aimed to achieve “herd immunity” —deliberately allowing citizens to become infected by the virus in order to create antibodies in the general population—in order to protect the economy and freedom of movement. However, while variations of herd immunity were implemented in the initial stage of the pandemic in countries such as the Netherlands (Holligan, 2020), Sweden (Henley, 2020) and the UK (Parker, 2020), they were later reversed. In contrast, none of the governments in East Asia implemented such a policy for their citizens.

Achieving herd immunity results in large numbers of infected people and deaths (D'Souza and Dowdy, 2020) which should not be the “ultimate goal” for controlling COVID-19 (Randolph and Barreiro, 2020). Further evidence shows that recovered patients can suffer from permanent damage to the lungs and other organs (Zhong et al., 2020) which is known as “long Covid” (Mahase, 2020).

Given Sweden's low population density, it suffered from one of the highest COVID-19 death rates in the world which reflected the failure of herd immunity. The elderly population were refused hospital treatment even when COVID-19 was in care homes, and this group accounted for 48.9 per cent of total deaths (Savage, 2020).

## Social Behavior

The East and West diverge in how individuals react to various pandemic measures, and this has contributed to different rates of spread through the population. Rather than Confucianism producing strict adherence to rules, its emphasis on respect for family and society (Tu, 1996) is what contributes to the difference. Thus, the communities of many East Asian regions worked together harmoniously and, for instance, did not object to waiting in long queues to buy facemasks. The Confucian notion of kinship is also reflected in the practice of sending facemasks from East Asia to friends and families across the globe. For example, during the shortage of PPE, a New York based journalist reported receiving facemasks from a Chinese friend in Beijing, and this trend was widely reported across the globe (Tett, 2020). Above all, citizens believed in the measures based on scientific evidence put in place to deal with the virus and acted (collectively) to reduce the transmission rate.

Some countries such as Italy and Spain also have a strong idea of familialism which prioritizes family values. However, individual freedom seems to be equally important because some Italian and Spanish citizens have not respected the rules for social distancing and wearing facemasks. Moreover, anti-lockdown protests have taken place in Italy (Deutsche Welle, 2020) and Spain (BBC News, 2020). The Italian Foreign Minister Luigi Di Maio called on the “coronavirus deniers to at least show respect for the families of the dead” (Deutsche Welle, 2020).

In May 2020, a number of anti-lockdown protests took place in the UK, Germany and the US over loss of civil liberties, despite the fact that breaking lockdown rules has contributed to the spread of the virus. Although some protests took place in China, they were small-scale, peaceful and social distancing was observed. And in contrast to Western countries, the protesters were small business owners who demanded landlords should reduce rents—they were not fighting for freedom of movement (Bloomberg News, 2020).

Certain Western countries such as Denmark (Milne, 2020b), Finland and Germany were praised for having successful pandemic strategies (Ben-Ami, 2020) due to low death rates in comparison with other Western countries. However, when comparisons are made with Eastern regions such as Hong Kong, Japan, Singapore, South Korea, and Taiwan, it is evident that the East has proven to be significantly more successful in managing the pandemic.

Problematically, the concept of public health has not been respected by some citizens in the West. This might be due to a public perception of the conflicts of interest in the close relationships amongst politicians, scientists and the pharmaceutical industry. Further, the MMR (Measles, Mumps, and Rubella) vaccine scare (Godlee et al., 2011), and the opioid crisis in Europe (Verhamme and Bohnen, 2019) and the US (The Lancet, 2017) have generated distrust of medical science and related fields of research. Profits are seen to be placed above the well-being of citizens. Perhaps raising the overall standard of science education could help the general public to understand scientific knowledge.

## Conclusion

This article highlights how East Asian governments made use of medical knowledge, public health expertise and social behavior to combat COVID-19. It further argues that a scientific state and competent population excelling in STEM subjects helps these societies in understanding and dealing with the pandemic.

In contrast, different pandemic strategies and versions of herd immunity were adopted for economic and social reasons (Fidler, 2020; Orlowski and Goldsmith, 2020) by some Western governments. This shows how politics can influence (Gonsalves and Yamey, 2020) the effectiveness of managing a pandemic in the West even though scientific knowledge from the East was available.

Above all, South Korea's COVID-19 management strategy has proven to work and the country subsequently reported an economic growth of 1.9 per cent in the second quarter of 2020, the sharpest rise in GDP in a decade (White, 2020). This shows that despite the current global pandemic, a competent government can lead a country out of a crisis.

When some Western countries experienced relatively lower numbers of COVID-19 cases, they were praised by commentators for their democratic and progressive ideology. However, when East Asia did extremely well in containing the virus, the West believed it was because East Asian countries are Confucian and draconian, and hence, obedient to undemocratic lockdown rules.

The anti-Asian sentiment and origin of the virus made it challenging for the West to seek pandemic solutions from the East. Perhaps the belief in Anglo-Eurocentric exceptionalism remains the West's biggest barrier to fighting COVID-19. This article highlights the importance of a constructive dialogue between experts which allows the exchange of the best pandemic strategies from across the world.

## Author Contributions

The author confirms being the sole contributor of this work and has approved it for publication.

## Conflict of Interest

The author declares that the research was conducted in the absence of any commercial or financial relationships that could be construed as a potential conflict of interest.

## Footnotes

^1^The US Centers for Disease Control and Prevention (CDC) publicly announced on 5 Oct 2020 that COVID-19 can be airborne, see https://www.cdc.gov/coronavirus/2019-ncov/more/scientific-brief-sars-cov-2.html.

^2^As of 31 October 2020, data obtained from: Our Word in Data, https://ourworldindata.org/coronavirus#coronavirus-country-profiles Johns Hopkins University Worldometer, https://www.worldometers.info.

^3^China (Beijing, Shanghai, Jiangsu, and Zhejiang), Hong Kong, Japan, Macau, Singapore, South Korea, and Taiwan.
